# Gel Formulations for Topical Treatment of Skin Cancer: A Review

**DOI:** 10.3390/gels9050352

**Published:** 2023-04-22

**Authors:** Marta Slavkova, Borislav Tzankov, Teodora Popova, Christina Voycheva

**Affiliations:** Department of Pharmaceutical Technology, Faculty of Pharmacy, Medical University of Sofia, 1000 Sofia, Bulgaria; btzankov@pharmfac.mu-sofia.bg (B.T.); tpopova@pharmfac.mu-sofia.bg (T.P.);

**Keywords:** skin cancer, physical hydrogel, chemical hydrogel, composite gel, systematic literature review

## Abstract

Skin cancer, with all its variations, is the most common type of cancer worldwide. Chemotherapy by topical application is an attractive strategy because of the ease of application and non-invasiveness. At the same time, the delivery of antineoplastic agents through the skin is difficult because of their challenging physicochemical properties (solubility, ionization, molecular weight, melting point) and the barrier function of the stratum corneum. Various approaches have been applied in order to improve drug penetration, retention, and efficacy. This systematic review aims at identifying the most commonly used techniques for topical drug delivery by means of gel-based topical formulations in skin cancer treatment. The excipients used, the preparation approaches, and the methods characterizing gels are discussed in brief. The safety aspects are also highlighted. The combinatorial formulation of nanocarrier-loaded gels is also reviewed from the perspective of improving drug delivery characteristics. Some limitations and drawbacks in the identified strategies are also outlined and considered within the future scope of topical chemotherapy.

## 1. Introduction

Skin is the largest organ in the human body, performing various functions ranging from protection to metabolism. The abnormal growth of the skin cells is referred to as skin cancer and this is the most common cancer type worldwide [1]. There are various types of skin cancer, including benign and malignant variants such as melanoma, squamous cell carcinoma and basal cell carcinoma [2]. Even though skin cancer is lethal in only 2% of cases in general, the malignant forms result in death in more than 80% of cases if not caught early [1]. The management of skin cancer includes surgical excision, radiotherapy, topical drug delivery, and oral therapy [2]. Although surgical treatment is considered the first line approach, topical therapy also has its role [3]. The most commonly prescribed medications are 5-fluorouracil [4,5,6] and imiquimod [5,7,8]. Other active pharmaceutical ingredients (APIs) are either repurposed or subjected to investigation and evaluation because of their topical chemotherapeutic potential in various forms of skin cancer. Some examples are doxorubicin [9], vismodegib [10], sonidegib [11], and metformin [12]. Substances of natural origin (curcumin [13,14], brucine [15], silymarin [16], chrysin [17], daidzein [18], and others) are also considered suitable in the topical treatment of skin cancer.

The topical dermal route provides ease of administration, non-invasiveness, and reduced systemic effects with the associated limited side effects and improved patients’ compliance [19,20]. Semisolid dosage forms occupy a serious part of the pharmaceutical formulations as they can be applied topically to the skin, cornea, nasal cavity, vagina, rectum, etc. Their specific rheological behavior governs their ability to adhere to the application site and prolong the API’s release. A significant advantage of these dosage forms is their ease of formulation and the possibility to incorporate various active moieties [21]. These advantages apply especially for gels which, in comparison to the other semisolid dosage forms, have higher retention time, provide excellent spreadability, and possess somewhat less long-term stability issues [22].

Nevertheless, there are some limitations arising from the properties of the API and the structure and function of the skin, and conventional gels cannot be a universal dosage form for treatment of skin conditions. In particular, if the cancerous cells are in a deeper layer of the skin, conventional semisolid formulations cannot provide adequate delivery of the chemotherapeutic [23]. Therefore, the combinatorial approach has been implemented in recent years to overcome these issues and improve dermal drug delivery. The nanoformulations solve major problems regarding the API solubility and penetration through skin layers, and they can guarantee controlled drug release and the possibility of delivering larger molecules or very hydrophilic compounds. However, their direct application is associated with probable compromised stability and limited retention time [24]. Thus, the increased number of publications researching the combinatorial drug delivery of nanocarrier-loaded gels is no surprise. A further possibility for improved therapeutic efficiency while simultaneously limiting the side effects in the adjacent skin tissues is targeted delivery of the API to the cancer cells [25,26]. The process can be either passive or active based on the underlying mechanisms involved. The passive targeting relies mainly on the enhanced permeation and retention effect (EPR). The tumor cells are characterized by excessive angiogenesis, which leads to the existence of considerably large gaps in the vascular walls. Together with the poor lymphatic drainage, it can be expected that nanoparticles of sufficient particle size (20–200 nm [25]) will permeate and be retained at the tumor site [27]. The active targeting is mainly related to the ligand functionalization of the nanoparticles. These surface-attached moieties can interact with the cancerous cells that overexpress receptors or proteins and lead to clathrin-mediated endocytosis and cell internalization [25]. Therefore, the active targeting not only enhances tumor accumulation but also increases intracellular delivery [28]. Possible overexpressed receptors that may be candidates for active targeting in skin cancers are folate receptor α isoform, transferrin receptor 1 (TfR1), CD44 surface markers for hyaluronic acid, and other proteins [25,26,28,29].

The aim of the current article is to systematically review the gel-based strategies for the therapy of skin cancer and to identify the main drawbacks and future aspects of gel development. The most commonly used excipients and their role are shortly discussed, and the most typical methods for characterization are given in brief. The current article summarizes the published approaches for gel-based formulations for the topical delivery of chemotherapeutics.

## 2. Systematic Search

### 2.1. Search Strategy

**Figure 1:**
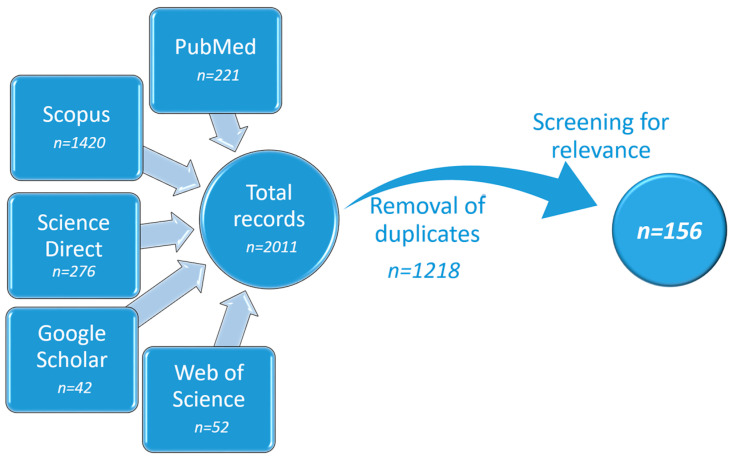
Flow chart of the systematic search strategy according to Page et al. [30].

The systematic review was conducting according to PRISMA guidelines [30]. The records were retrieved after searching in major scientific databases, namely, Google Scholar, PubMed, Scopus, ScienceDirect, and Web of Science. The search was limited to the title, abstract, and keywords without the full text. The year of publication was limited up to 2022 inclusively. The terms searched were identical in all databases (“skin cancer” AND “gel” OR “hydrogel” OR “nanogel”). The duplicates were removed from the identified papers with the help of Zotero software (v. 6.0.22), and the rest were browsed for relevance. The search strategy, flow diagram, and retrieved articles are presented in Figure 1.

### 2.2. Inclusion and Exclusion Criteria

Only research articles were considered relevant while all therapeutic guidelines for medicinal practice, clinical guidelines, conference proceedings, book chapters, therapeutic strategies, and updates, as well as all review papers, were excluded. Papers that do not address the potential gel application as a topical dosage form in skin cancer therapy were also excluded. Articles published in 2023 were not included in the search. No language limitations were used in the current search strategy.

All papers considered as relevant (*n* = 156) were original articles discussing the development, evaluation, and/or characterization of gel formulations used in the potential topical treatment of skin cancer.

## 3. Drug Delivery Hurdles in Skin Cancer Treatment

Chemotherapy of skin cancer can be either oral, parenteral, or topical. In the first case, only a limited amount of the drug reaches the target site, while the rest reaches other organs, tissues, and cells, and can cause harmful side effects. The case is quite similar in parenteral application together with its invasiveness. Therefore, the local application on different skin cancer forms can gain in therapeutic efficacy and safety. However, there are numerous obstacles for drug penetration, which makes the topical delivery a challenging task.

### 3.1. Skin Structure

**Figure 2:**
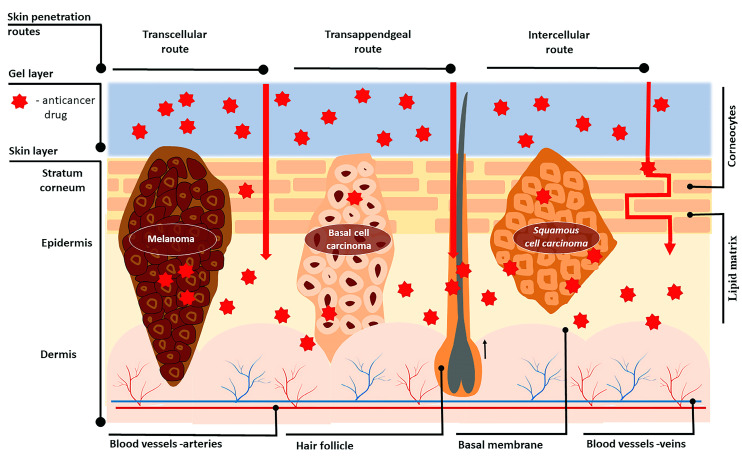
Skin structure and routes of drug transport through cancerous skin after topical anticancer treatment.

Skin is a complex organ with the main function to “maintain the insides in and outside out”, acting as a barrier [31]. It consists of three layers—the epidermis, dermis, and hypo-dermis—each having a different composition and properties [32] (Figure 2). The inner hypodermis consists of adipose tissue and rarely plays an important role for drug delivery [31]. The dermis (around 2–4 mm) [33] is built up by a collagen and elastin network in a mucopolysaccharide gel which resembles a hydrogel structure. The vascularity of the dermis enables the transport of the most transdermally delivered drug molecules into the blood, maintaining a high concentration gradient. This layer also includes appendages—hair follicles, sebaceous and sweat glands—which have an impact only on the initial diffusion through the skin [31]. The outermost skin layer—the epidermis—consists mainly of cells, called keratinocytes, which play a lead role in topical drug delivery [34]. The epidermis has two avascular and hydrophobic sections, the viable epidermis and the stratum corneum (SC). The viable epidermis consists of four layers of keratinocytes at different stages of differentiation, melanocytes, Merkel cells, and Langerhans cells. The differentiation of the epidermis starts from inside to outside, and results in the formation of corneocytes—dead, anuclear, flattened, and keratin-rich cells [33]. The corneocytes are surround by lipid matrix composed of triglycerides, cholesterol, free fatty acids and ceramides, and these structures act as the prime barrier to the entry of macro and micro anticancer molecules across the skin, limiting drug delivery [35,36,37]. Normal melanin cells are characterized with physiological pH while tumor cells have a pH in the range 5–6.5 [38]. In the case of melanoma, the acidic pH is responsible for the invasion of surrounding tissues and the malignant cells have higher metastatic capacity [39]

Moreover, skin cancer studies revealed there are higher levels of keratin and lipids in cancer cells compared to healthy cells, resulting in a thicker SC layer and consequently a stronger barrier to drug entry, which makes it even more difficult for anticancer molecules to reach the tumor site [34,40]. This is why transdermal drug dosage forms, employed against actinic keratosis (AK) and basal cell carcinoma (BCC), require proper design to reach the deeper epidermal layers [32,41,42]. The optimal particle size needed to achieve transdermal delivery lies in the range 200–400 nm [43]. Nanoparticles with sizes of about 300 nm can reach deeper skin layers through the transappendgeal route [44].

### 3.2. Skin Penetration Routes and Factors Influencing Skin Penetration

The penetration of drugs through the skin can happen by three possible routes depending on the physicochemical properties of the active substance: intracellular (through the stratum corneum), intercellular (through the lipid matrix), and through the skin appendages (sweat glands or hair follicles) [31] (Figure 2). The transport via skin appendages (shunt route) is more suitable for hydrophilic molecules, but since the fractional area of appendages is relatively small, this shunt route is not as important for drug delivery [45]. On the other hand, highly lipophilic drug molecules can easily pass through the skin intercellularly via the lipid matrix. The intercellular (transcellular) route is the most complicated one because the drug molecule should repeatedly pass through different media of the “brick wall” [46]. First, the permeant should partition into keratin-filled corneocytes (hydrophilic environment), and after that it should diffuse through the corneocytes, followed by partitioning into the intercellular lipid matrix (lipophilic environment) [47]. Since crossing the lipid bilayers is involved in both transcellular and intercellular routes, diffusion through these lipid media is highly important. Therefore, lipophilic drugs are preferred candidates for transdermal delivery [31]. However, when passing through the stratum corneum, molecules reach the more hydrophilic lower epidermal layers (viable epidermis), and in the capillaries of the epidermal–dermal junction, they can be cleared, entering the systemic circulation [41]. Thus, high lipophilicity hinders the clearance. Ideally, the permeant should possess moderate hydrophilic–lipophilic properties, expressed as a logarithm partition coefficient (log P_water/octanol_) in the range 1–3 [32,45].

Unfortunately, not all anticancer medications possess this desired hydrophilic–lipophilic balance. For example, 5-fluorouracil, approved for actinic keratosis (AK) and superficial basal cell carcinoma (sBCC), is a highly hydrophilic molecule (log P: −0.89) [48]. This hydrophilicity restricts penetration through the hydrophobic stratum corneum and is reflected in low treatment efficacy for deeper laying lesions [48]. Furthermore, the insufficient skin penetration of 5-FU requires frequent and higher administration doses, which leads to side effects such as skin inflammation [49,50,51]. The situation with imiquimod, used for the treatment of BCC, is similar. Due to its low water solubility, permeability within the hydrophilic dermis media is difficult. Moreover, there is an interaction between the amine groups of the drug molecule with the anionic components of the skin, limiting additional imiquimod permeation, and resulting in reduced therapeutic effect [52,53].

Another factor that influences skin penetration is the molecular weight of a permeant as the transport of molecules via the skin happens by passive diffusion under a concentration gradient, following Fick’s law. According to the Stokes–Einstein equation, the diffusion coefficient of a molecule increases with the increase in its approximate radius. Therefore, a higher molecular weight is related to a higher approximate radius, so that the diffusion coefficient is generally smaller and thus the diffusion is hindered. For transdermal delivery, the drug’s molecular weight (MW) should be less than 500 Da [31,32], making the penetration of anticancer drugs with higher molecular weight difficult.

Another problem arising in cancer therapy is multidrug resistance (MDR). The interaction between the drug and the tumor media is a complex phenomenon and cancers can exhibit significant resistance to various molecules. Multidrug resistance can be defined as the decrease in the efficacy and potency of a drug to produce a therapeutic effect and is a major problem that reduces the chemotherapies’ effectiveness [34,54]. Drug resistance in skin cancers can be primary (intrinsic) or acquired. Primary resistance appears without prior exposure to anticancer agents, and thus the initial response to treatment is poor [55]. Acquired resistance is developed during the application of the cytostatic drug, and it is associated with devastating results after initially good ones [54,56].

Different mechanisms are associated with intrinsic resistance, such as changes in drug transport and efflux pump, alteration in enzyme activation and DNA repair, modulation of the apoptotic pathway, etc. [56,57,58,59]. Acquired drug resistance is affected mainly by genetic or environmental factors that enable the progress of drug-resistant cancer cell lines or induce enzyme mutations [54,55,60]. Therefore, understanding the modifications in molecular processes involved in drug resistance can trigger the development of new therapeutic strategies against skin cancers.

One of the potential factors leading to the sensitivity of drugs is the limited amount of drug reaching the tumor cells. This is why the determination of the “maximum tolerated dose” (MTD)—the highest single dose of an agent that does not cause significant or intolerable toxicity effects—is of great importance [54,61]. Therefore, any methods for increasing the penetration of the anticancer drug through the skin, and thus increasing the amount reaching the tumor cells, can lead to improved therapeutic outcomes.

### 3.3. Opportunities for Increasing Skin Penetration

Considering all the issues discussed and the factors involved in managing the transport of anticancer drugs through the skin, the most important challenge of therapy is the improvement in the drug uptake, allowing the drug to pass through the deeper layers of skin, inside cancerous cells [32].

One of the techniques to augment penetration is the utilization of “penetration enhancers” [32,62], such as ethanol, Azones, fatty alcohols, glycols, and DMSO. These substances mainly disrupt the lipid bilayer packing, interacting with intercellular proteins [31]. It is important to know that water possesses enhancer properties, as the drug diffusion is higher through hydrated skin; thus, occlusion is necessary for improved therapy [62].

Many dosage forms are used for topical delivery of skin anticancer medication, such as powders, aerosols, emulsions, and creams. However, hydrogels have superior properties [32,63]. Their structure allows them to be controlled at a molecular scale, which can be used to modify the properties such as degradation rate, long-time release, tunable pore size, and chemical and biological response to stimuli such as pH, enzymes, and temperature, in accordance to desired values [64]. Moreover, hydrogel-based drug dosage forms exhibit improved chemotherapy outcomes by increasing drug half-life, enabling controlled drug release, and reducing nontargeted exposure [65,66,67].

The combination of gel formation technology and nanotechnology leads to the creation of advanced drug delivery systems such as nanogels, liposomes, ethosomes, niosomes, and transferosomes, improving the skin penetration and bioavailability, and can be potentially used for topical skin cancer therapy [40,49,68]. These strategies either alone or in combination are discussed in the following sections of the current review.

## 4. Gel-Based Formulations

According to European Pharmacopoeia, gels are a class of semisolid preparation for cutaneous application, which consist of liquids gelled by means of a suitable gelling agent [69]. They possess a three-dimensional network due to the formation of covalent or noncovalent bonds of the polymer used with the medium [70]. Based on the polarity of the liquid, the Pharmacopoeia further classifies them into hydrophilic and lipophilic gels [69]. There are a large number of other criteria in the literature, based on which the gels can be distinguished: nature of the solvent, colloidal phases, rheology, the origin of the gelling agent, etc. Three main types can be classified based on the nature of crosslinking, namely, physical gels, covalently cross-linked gels and entanglement network gels [70]. This review uses these three classes of gels in order to discuss their properties and application in chemotherapeutics delivery in skin cancer. Physical gels can be prepared with natural, synthetic, and semi-synthetic polymers such as carbomers, gelatin, marine polysaccharides, and plant polysaccharides. Typically, these gels use temporary cross-linking and therefore they are transient in nature. The second group are the covalently cross-linked gels, which are also referred to as chemically cross-linked gels. In their structure, chemical bonds exist between the macromolecules and the cross-linker and these bonds are susceptible only to thermal degradation. These gels are characterized with high elasticity due to the larger volume of solvent and the flexibility of the polymer chain. Polymeric hydrogels are a subgroup in this class prepared by cross-linking a three-dimensional hydrophilic polymeric network swollen in water. In the last class (entanglement networks), gels are formed when the concentration and molecular weight of the polymer used exceed the critical molecular mass of entanglement. Otherwise, they form dilute polymer solutions [70].

**Figure 3:**
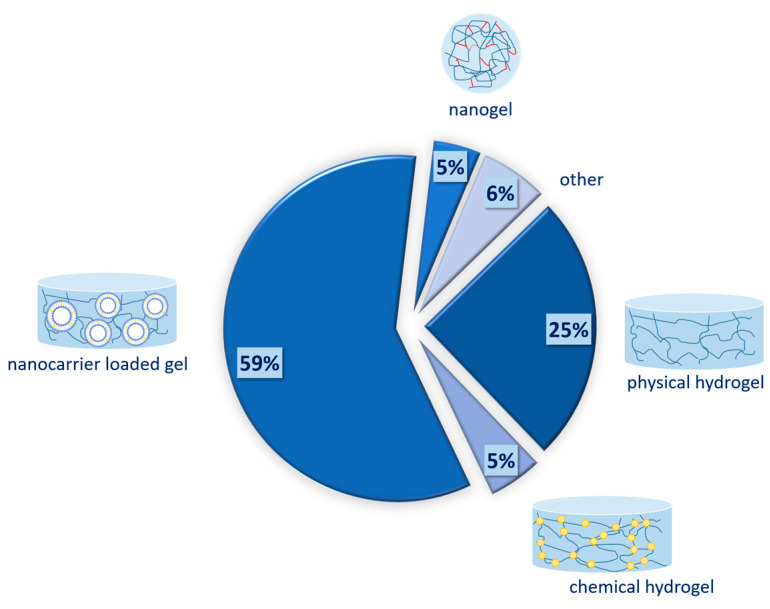
Types of gels used for topical drug delivery in skin cancer treatment according to the systematic search.

The systematic search retrieved 156 relevant records, as can be seen in Figure 1. The distribution of papers between the different types of gels can be seen in Figure 3. Articles that discuss the preparation and characterization of physical hydrogels comprise 25% (*n* = 39). None of the retrieved records discusses the preparation of organogels. This is probably due to the advantages of hydrogels, such as their ease of preparation, non-greasy nature, and cooling sensation upon application [71]. Chemically cross-linked hydrogels account for 5% (*n* = 8). Even though chemical hydrogels are extensively studied in chemotherapy, their primary application is parenteral. Therefore, the current search provided only limited articles. Nanogels, which are characterized by small particle sizes in the range of 20 to 250 nm, are subjects in about 5% of articles (*n* = 7). Nanocarrier-loaded gels are either physical or chemical hydrogels in which different nanocomposites are incorporated. As can be seen on Figure 3, they represent the majority of the identified articles, 59% (*n* = 92), due to the combined and versatile properties they provide. In the group of others belong gels that do not fall into any of the previous categories, such as bigels and emulgels, or that are unclear due to unavailable full text. In the following sections, each of those types of gels is discussed in brief within the scope of topical dermal delivery in skin cancer therapy.

### 4.1. Physical Hydrogels

Physical hydrogels are those types of dosage forms in which water with or without other polar liquids is used as the medium and the gelling agent is physically cross-linked due to electrostatic interaction, ionic interchain bridges, crystallization junctions, hydrophobic association, hydrogen bonds, or others [72]. The most typical characteristic for these gels is their reversibility and the temperature-dependent sol–gel transition. Additionally, the polymers used in their preparation possess a good safety profile as they are nontoxic, biocompatible, and non-reactive, and no residual cross-linkers can be found in comparison to chemical hydrogels [70,73]. This section of the review discusses the application of various natural, synthetic, and semi-synthetic polymers suitable for the preparation of physical gels with chemotherapeutic agents for skin cancer.

#### 4.1.1. Carbomer as a Gelling Agent

Carbomers are synthetic high-molecular-weight polyacrylic acid derivatives cross-linked with allyl sucrose or allyl pentaerythritol. They are anionic in nature and contain between 56 and 68% *w*/*w* carboxylic acid groups [74,75]. They are capable of forming hydrogel bonds with mucin, and therefore possess bioadhesive properties [75]. Commercially, carbomers are available under the trade name Carbopol^®^. The main difference between the various types of carbomers is based on the differences in their production and, therefore, the resulting properties such as solution viscosity. For example, Carbopol^®^ 934, 940, and 941 are prepared by polymerization in benzene as a solvent, while the newer NF (National Formulary, United States Pharmacopoeia) grades are synthesized in a benzene-free process. Other variations could be due to the type and amount of cross-linker used [76]. The technological procedure of carbomer gel preparation is well-established and is based on the hydration of the polymer in water, resulting in an acidic dispersion with pH = 2.5–3.5 depending on carbomer type and concentration [77]. Then, neutralization with sodium hydroxide, triethanol amine, or other base leads to ionization of carboxylic functional groups, followed by partial disentanglement of polymer chains and formation of irreversible agglomerates [78,79]. Very often the medium of carbomer gels contains, in addition to water, polyethylene glycol (PEG 400) or glycerol, which may affect the rheological and mucoadhesive properties [78]. In the current review, Carbopol^®^ 934 was found to be most commonly applied in order to prepare conventional physical gels. The concentrations ranged from 0.5% to 3% *w*/*w* [80,81,82,83,84].

One of the drawbacks in carbomer-based physical gels is the aqueous medium in which they are prepared. On one hand, this raises the need for the addition of other excipients such as preservatives [85] in order to prolong microbial stability. On the other hand, there is a significant number of drugs that are not water soluble such as resatorvid, which is used to treat skin cancer. This requires the addition of a suitable co-solvent as suggested by Ruiz et al. [80]. Another work group of Osipitan et al. [81] compared phenethyl isothiocyanate (a phytochemical, PEIT) formulated in 0.5% Carbopol^®^ 940 to an analogical gel containing 5-fluorouracil. The incorporation of the PEIT similarly required its dissolving in DMSO prior to the gel preparation. DMSO can further potentiate the effect because it is commonly used as a penetration enhancer. The conducted investigation showed also that it is compatible with carbomer gel [81,84]. Recombinant heat shock protein (Hsp70) was incorporated into 1% carbomer in the presence of 1% glycerol and 10% DMSO by Abkin and colleagues [82]. Their investigation showed that the protein was prevented from undergoing denaturation and/or proteolysis during storage. The lyophilized methanolic root extract of *Annona reticulate* was successfully incorporated into Carbopol^®^ 940 gel (1% *w*/*w*) by Bharadwaj et al. with the help of 10% glycerol [83]. The authors showed the potential of the formulation for dermal anticancer treatment.

Photodynamic therapy (PDT) could also benefit from the application of physical gels. Non-toxic chemicals called photosensitizers are applied either orally or locally to produce visible fluorescence when activated through illumination with an appropriate wavelength [86]. A classic example of a drug used in PDT is porphyrins and their precursor, 5-aminolevulinic acid (5-ALA). Even though 5-ALA is a small molecule, its hydrophilic nature hinders penetration through the stratum corneum. A possible approach to resolve the issue is via its chemical modification as a methyl or hexyl ester. An alternative could be the use of penetration enhancers such as DMSO [87]. Merclin and co-workers investigated the possibility of using carbomer physical gel containing 5-ALA or methyl-ALA by means of iontophoresis [84]. Since hydrogels serve as contact gels between the skin and electrodes in iontophoresis, they could be used as a vehicle to deliver APIs to the deeper skin layers. Furthermore, carbomer is an anionic polymer and possesses good buffering capacity, which can be beneficial in maintaining constant pH. Interestingly, the authors investigated this and showed that the diffusion of uncharged drug in 1% w/w carbomer gel is similar to that in water. At the same time, positively charged drugs are retained to some extent [84].

The given examples of carbomer-based physical gels show that this is a versatile platform for the direct loading of hydrophilic and lipophilic substances, which can be applied in combination with phototherapy, radiotherapy, and iontophoresis. Nevertheless, the limitations to deeper penetration of the APIs into the skin layers still exist, which in these types of gels depend significantly on the properties of the drug itself. Therefore, physical gels produced by means of carbomer are suitable semisolid vehicles for the treatment of surface skin cancer given suitable drug solubilization is achieved.

#### 4.1.2. Cellulose Derivatives as a Gelling Agent

Different cellulose derivatives have been exploited in the preparation of various hydrogels and there are already published reviews discussing their properties, preparation techniques, and fields of application [88,89,90]. The current article focuses on their application as a dosage form for topical chemotherapeutic delivery.

Cellulose is a polysaccharide obtained from various plants, animals, or bacteria. It is renewable and abundant worldwide, which together with its biodegradability, biocompatibility, environment friendliness, and good mechanical properties, makes it suitable for numerous areas of application. Cellulose is a semicrystalline linear polymer of glucose that is tasteless and odorless but insoluble in water, like many organic solvents [88]. Its aqueous insolubility hinders the biomedical application of cellulose. Therefore, chemical modification of its hydroxyl group through etherification and esterification leads to the preparation of substances with improved solubility [91]. Many cellulose derivatives are used in the development of the vast majority of drug delivery systems including liquid, semisolid, and solid conventional dosage forms [92] and the relatively newer nanocomposites [93]. In addition, the cellulose derivatives can form physical hydrogels due to hydrogen bonds, ionic interactions, and hydrophobic forces [90].

Hypromellose or hydroxypropylmethyl cellulose (HPMC) is an electroneutral water-soluble derivative of cellulose to which methyl and hydroxypropyl side groups are attached. The polymer provides a wide range of viscosity grades depending on the substitution degree and ratios [94]. The systematic search retrieved four articles considering HPMC as a gelling agent in physical hydrogels [95,96,97,98]. Two of them investigated the incorporation of cyclodextrin complexes with APIs into a semisolid gel. Ceschel and co-workers showed that the HPMC-based gel has its own solubilizing effect, as the release rate from a gel containing the API and a gel containing the API-cyclodextrin complex is identical. Nevertheless, the cyclodextrin complex showed better permeation. The authors hypothesized that this is due to the presence of a diffusion layer on the skin surface and the cyclodextrin acts as a carrier through it towards the lipophilic skin layer [95]. In another more recent study performed by Doneda and colleagues, different cyclodextrin complexes of the lipophilic flavonoid 3-*O*-methylquercetin were used to allow its incorporation into a hydrophilic topical formulation [97]. HPMC was used as a gelling agent at 3.5% concentration in the presence of 1% propylene glycol. All of the proposed gels showed suitable characteristics for dermal application such as pH in the range of 4.49 to 4.87 and pseudoplastic rheological behavior. Bioadhesion was also established for the formulations even though the presence of β-cyclodextrin or hydroxypropyl-β-cyclodextrin complex leads to some reduction in their bioadhesive capacity due to interaction with HPMC.

Carboxymethyl cellulose (CMC) possesses carboxylate moiety which is pH-sensitive and provides the polymer with in situ gelling properties as well as bioadhesive ability [99]. However, physical gels prepared by means of CMC are characterized by low mechanical stability. Except by chemical cross-linking, this issue can be overcome by the addition of naturally available nano-clays such as sepiolite. Palem et al. used simple moisture heat treatment to prepare a physical gel of CMC, agar, PVP, and sepiolite. The platform was non-toxic and showed stable and sustained release of 5-fluorouracil.

Another cellulose derivative used in the delivery of 5-ALA or glycoalkaloids is hydroxyethyl cellulose (HEC) in the concentration range of 2–3%. HEC is non-ionic cellulose ether that is water-soluble [94]. A study performed by Maisch et al. showed that the depth of 5-ALA penetration from 3% HEC gel containing 40% DMSO as an absorption enhancer was higher in comparison to hydrophilic ointment, lipophilic ointment, or w/o gel containing DMSO. Nevertheless, low chain alcohols were shown to act better as penetration enhancers in the case of 5-ALA. Its solution in the mixture of ethanol, isopropanol, and polyethylene glycol with tetraethylene glycol ether is superior to the newly proposed physical gel in terms of penetration. Tiossi and co-workers used commercial 2% physical hydrogel of HEC to deliver glycoalkaloids from *Solanum lycocarpum* fruits for the treatment of non-melanoma skin cancer [100]. In order to promote skin permeation, different penetration enhancers were used and it was proven that 5% monoolein, alone or in combination with 10% ethanol, is most suitable to achieve permeation to the target site. These absorption enhancers are compatible with HEC gel and could be used for the delivery of other chemotherapeutics.

The results shown in the discussed articles suggest that cellulose derivatives alone are not ideal as a delivery platform for chemotherapeutics in the treatment of skin cancer. There is a need for the addition of excipients such as cyclodextrins or penetration enhancers in order to achieve the desired depth of penetration.

#### 4.1.3. Poloxamer as Gelling Agent

Poloxamers known by their trade name Pluronics (BASF, Ludwigshafen, Germany) are a group of synthetic triblock copolymers of poly(ethylene oxide) (PEO) and poly(propylene oxide) (PPO) in the arrangement [PEO]_n_-[PPO]_m_-[PEO]_n_, with varying numbers of monomers (n and m) from each block [101]. They are thermoresponsive and their micellization, aggregation, and gelation depend on temperature [102]. The micellization is due to the dehydration of PPO blocks, which are then located in the core and the hydrated PEO chain forms an outer shell [103]. The critical gelation concentration and gelation temperature of poloxamers decrease with the increase in their molecular weight. Poloxamer 407 (Pluronic^®^ F127) is known to form a reversible gel at a concentration above 20% and temperature of about 19 °C. The higher the polymer concentration, the lower the gelation temperature will be [86,104]. One of the first investigations of poloxamer gel for dermal delivery of 5-fluorouracil or doxorubicin dates back to 1984 [105]. The authors found that the apparent release of the APIs from the gels depends on the concentration of the gelling agent. The released amount was improved with an increase in the temperature of the in vitro dissolution test. These findings suggest that the release happens through the aqueous channels around the polymer micelles. The more polymer is used for gelling, the smaller those channels are, while with the increase in temperature the micro-viscosity in the channels decreases. Redpath and co-workers found that 30% blank poloxamers 407 gel without API being loaded slightly inhibited the cell migration in C8161 melanoma cell lines without affecting the cell viability, but did not further investigate the observation. Furthermore, their study showed that the release of silybinin from the gel is somewhat retarded due to the slow diffusion of the drug in the gel and the diffusion is the rate-limiting step. The gel crystalline structure is less permeable than the liquid crystalline one and the results showed that Poloxamer 407 gel had limited drug flux into the skin. The polymer did not affect the skin’s lipid integrity and lower penetration was observed [104]. Another study investigated the possibility of forming an in situ gelling formulation consisting of 7% poloxamer 407 and 40% poloxamer 188. In situ gels possess several advantages, such as easy application simultaneous with prolonged adhesion on the skin surface and good permeability of therapeutic agents. Nevertheless, the investigation of Sun and colleagues showed that for lipophilic APIs such as curcumin the release rate was limited by the drug dissolution. Applying the cyclodextrin complex significantly improved the potential to effectively deliver curcumin to melanoma cells [103].

Batista and team investigated the stability of a poloxamer 407 gel in the case of incorporation of hydroalcoholic extract. Evidently, the gelling agent is compatible with ethanol in comparison to HEC-based gel [106]. Nevertheless, poloxamer 407 gels possess some disadvantages such as low mechanical strength, low durability, and very fast drug release [107]. As shown by Sun and co-workers, the erosion of in situ gel based on both poloxamer 188 and 407 was about 40% within 3 h [103]. Therefore, it is common to blend poloxamers with other gelling polymers. In the current review, mixtures of poloxamers and carbomer were identified for the topical delivery of anticancer drugs [86,108]. The addition of carbomer could improve the stability and consistency of the poloxamer gel and further provide it with bioadhesive properties. This could prolong the contact time and improve the drug delivery. Borghi-Pangoni and colleagues showed that the required concentration of a combined poloxamer/carbomer gel is 20%/0.15%, respectively, in order to achieve appropriate in situ gelation without runoff upon application. The poor aqueous solubility of hypericin could be overcome to some extent in the proposed gel. Even though a rapid release of the drug was present (within 2 h), no permeation was evident. Therefore, the proposed dosage form could be suitable for photodynamic therapy [86]. The study of Campanholi and colleagues also supports these conclusions. The authors showed the solubilizing capacity of the combined poloxamer 20% and carbomer 0.2% gel for chlorophyll used in photodynamic therapy [108].

Poloxamer gels could be used in the chemotherapy of skin cancer, providing that suitable measurements are taken in order to improve the gel stability and solubilization of lipophilic APIs. Furthermore, prolonging the contact time by adding bioadhesive polymers such as carbomer could further positively affect the properties of such drug delivery systems. Otherwise, the drugs are superficially released without penetration. This could be desirable in the scope of photodynamic therapy.

#### 4.1.4. Other Physical Hydrogels

Chitin is another polysaccharide which forms physical hydrogels upon desolvation or physical cross-linking [109]. Chitin is a natural co-polymer of glucose and acetamide. In the case where the degree of acetylation is lower than 50%, the biopolymer is called chitosan. The formation of the physical hydrogel by means of chitin first requires its dissolving in a suitable solvent as it is not water-soluble. The increase in the gelling agent concentration leads to more pronounced entanglement and an increase in the hydrogen bonding, followed by a transition from sol to gel. Furthermore, the stability of the hydrogel can be increased by curing at various temperatures or by coagulation in a specific antisolvent. The antisolvent approach was used by Nair and co-workers in order to prepare a 0.5% chitin hydrogel loaded with plumbagin [110].

Another natural polymer found to form physical gels is iota-carrageenan. The 3D structure formation is due to the aggregation of the macromolecules through electrostatic interactions and hydrogen bonds. Successful loading of methotrexate cyclodextrin inclusion complex into 1% iota carrageenan was presented by Kochkina et al. [111]. The cyclodextrin complex did not influence the gel rheology and mechanical stability.

The literature search provided the information that scientific efforts have been made to synthesize novel polymers that can form physical hydrogels and deliver lipophilic APIs to skin cancerous cells. Such is the case with the synthesized ABA type cationic triblock polymer developed by Taktak et al. [112]. The amphiphilic nature of the polymer and its positive charge could lead to interaction with the stratum corneum by promoting the penetration of lipophilic drugs such as paclitaxel.

### 4.2. Chemical Hydrogels

Covalently cross-linked hydrogels are 3D swollen networks which are prepared by hydrophilic polymers containing various functional groups, such as -OH, -SO_3_H, -COOH, -CONH-, and -CONH_2_. These groups are either embedded or grafted in their structure, allowing them to absorb and retain large amounts of water and biological fluids. They do not dissolve in water but rather swell and hold up the medium [70]. Their structure can be tuned by varying the degree of chemical cross-linking and the affinity of the hydrogel towards the aqueous medium [9]. Examples of chemical hydrogels investigated for the treatment of skin cancer are given in Table 1.

Carboxymethyl cellulose (CMC) can be efficiently cross-linked with citric acid in order to prepare biocompatible hydrogel with no cytotoxicity [9,113,114]. A prodrug conjugate between CMC and doxorubicin was prepared and afterwards cross-linked with the acid [9,114]. The study shows promising sustained release of the drug as the covalent bondamide bonds will be cleaved predominantly in the lysosomes after cellular uptake in the melanoma cancer. The authors further explored the possibility of preparing hybrid hydrogel containing CMC-silver nanoparticles electrostatically associated with the same API and further cross-linked with citric acid to form the gel [113]. This newly proposed formulation showed synergistic anti-tumor effect between doxorubicin and the silver nanoparticles and in addition possessed antibacterial activity.

Oktay and Alemdar prepared an electro-responsive hydrogel based on gelatin for stimulus triggered release of 5-fluorouracil [115]. Gelatin was modified with methacrylic anhydride and further subjected to UV cross-linking. They encapsulated a conductive polymer which controlled the drug release, and the application of 1.5 V led to anisotropic gel deformation and the amount of 5-fluorouracil was sufficient for the treatment of skin cancer.

**Table 1:** Examples of APIs formulated in chemical hydrogels and the corresponding excipients used.

Polymer	Cross-Linker	API	Method of Preparation	Ref.
Gelatin	Metacrylic anhydride	5-fluorouracil	UV cross-linking	[115]
CMC	Citric acid	Doxorubicin	“green” method	[9,114]
	Citric acid	Doxorubicin	“green” method	[114]
	METAC ^1^, DEGDMA ^2^	curcumin	Free radical polymerization	[116]
Carboxymethyl chitosan	Glutaraldehyde	5-fluorouracil	Gelled deoxycholic acid micelles	[117]
Poly(acrylamide-co-diallyldimethylammonium chloride)	BisAA ^3^, APS ^4^/TEMED ^5^	Indocyanine green	Free radical polymerization	[118]
Chitosan	PEGDA ^6^	*Aloe vera* juice	UV cross-linking	[119]

^1^ METAC-[2-(metacryloyloxy) ethyl] trimethyl-ammonium chloride; ^2^ DEGDMA-diethylene glycol dimethacrylate; ^3^ BisAA-N,N’-methylenebisacrylamide; ^4^ APS- ammonium persulfate; ^5^ TEMED-N,N,N′,N′-tetramethylethylenediamine; ^6^ PEGDA-diacrylate poly(ethyleneglycol).

A hydrogel-based transdermal system for the potential treatment of skin cancer and radiotherapy-induced burn wounds was prepared and characterized by Kudłacik-Kramarczyk et al. The formulation was based on chitosan and cross-linked with PEGDA (diacrylate poly(ethyleneglycol)). Chitosan hydrogels need an alkaline medium to coagulate and provide the characteristic rheological behavior. Their strength can be improved by ionic cross-linkers such as sodium citrate or tripolyphosphate, which also provide them with pH sensitivity [109]. The authors performed intensive physico-chemical characterization of the prepared gel and proved its biocomparibility.

The hydrophilic properties of the hydrogels significantly limit their ability to deliver hydrophobic drugs. In such a case, the loaded amount is lower and unevenly distributed in the gel structure, and shows inadequate release. In order to overcome this issue, Pourmanouchehri et al. proposed incorporation of desoxucholic acid micelles containing 5-fluorouracil in a hydrogel. Carboxymethyl chitosan cross-linked with glutaraldehyde was shown to be less soluble at pH = 9 in comparison to the unmodified polymer [117]. Therefore, the authors found a better and more complete release of 5-fluorouracil in an acidic medium (pH = 6.8 and pH = 5). Furthermore, the prepared hydrogel prevented deoxycholic acid micelles’ destabilization and had no burst effect. Altogether, these measurements improved the cytotoxic effect of the API five-fold compared to free 5-fluorouracil and proved the efficiency for the treatment of melanoma

Hwang and Jin synthesized a polymer for the preparation of a chemical hydrogel which can be loaded with indocyanine green and used in combination with photodynamic treatment of melanoma [118]. This type of skin cancer is especially difficult to treat due to its fast spreading to the adjacent skin tissue. The produced hydrogel is mechanically stable and can be attached to the skin. The proposed formulations appear to be superior for the PDT in comparison to an injectable hydrogel, as they offer irradiation protection of the loaded indocyanine green and exert less irritation at the site of application. The authors demonstrated complete elimination of the melanoma cells in the tested mouse model.

A different strategy for topical antineoplastic drug delivery by means of chemically cross-linked hydrogel has been proposed by Oktay and Alemdar [115]. The authors prepared electro-responsive hydrogel loaded with 5-fluorouracil. Gelatin was modified with methacrylic anhydride and further subjected to UV cross-linking. They encapsulated a conductive polymer which controlled the drug release and the application of a 1.5 V electric current led to anisotropic gel deformation. Thus, the released amount of 5-fluorouracil was sufficient for the treatment of skin cancer.

The limited number of articles in this category of hydrogels is possibly due to the fact that they usually do not possess the typical rheological properties for topical application. Thus, they are applied as plasters or after being injected directly into the cancerous cells [120].

### 4.3. Nanogels

Nanogels are a category of hydrogels characterized by a 3D porous structure and particle size in the range from 20 to 250 nm [121]. Other definitions put nanogel particles in the range from about 10 nm to 1000 nm [38]. Similarly, they can be either physically or chemically cross-linked. The second can be prepared either by polymerization of monomers or by cross-linking of preformed polymers [122]. In comparison to typical nanoparticles, nanogels possess tunable particle size and particle shape, and sensitivity to various external stimuli (pH, temperature, ionic strength, etc.) [121]. Therefore, they mostly present drug delivery carriers for systemic therapy. Nanogels can also be used for topical delivery, as their unique structure and mechanical properties resemble those of the skin’s extracellular matrix [14]. However, this route of administration encounters a major hurdle, which is the limited drug diffusion across the stratum corneum together with the possibility of spilling and aggregation over time [14,38]. Therefore, surface modification is often needed in order to overcome these obstacles [38,43,123]. Examples of such formulations can be seen in Table 2.

Transcutol^®^ (di-ethylene glycol monoethyl ether) can interfere with cell membranes in the stratum corneum and thus acts as a penetration enhancer. It can be added to simple gel formulations in concentrations ranging from 1 to 50% and promotes drug permeation [128]. The usual concentration of 22% *v*/*v* guarantees diffusion through the skin’s lipid matrix. Combining poloxamer 407 with Transcutol^®^ results in a stable nanogel formulation and avoids the irritation potential of the penetration enhancer [123]. Another strategy to limit irritation, edema, and inflammation is the application of natural penetration enhancers, as suggested by Sahu et al. [38]. Such substances are terpenes and terpenoids, constituents in eucalyptus and chenopodium essential oils. They can significantly improve the penetration depth and therefore increase the therapeutic potential of the formulation.

Chitin and chitosan-based nanogels showed improved drug release in an acidic environment (pH = 4.5–6), which is very suitable for the skin delivery of APIs. The main reason for this phenomenon is the possible protonation of free amino groups in the polymer molecule [43,123,124,125,126]. This is additionally helpful in regard to the possibility of ionic bonding with the tumor cells because of their acidic nature (pH = 5.5–6.5), and thus release in a controlled manner can be expected [123].

An alternative approach has been proposed by Priya et al. [14]. In the study, a layer-by-layer technique is applied. First, self-assembled CMC-casein nanogel as a core is formulated and loaded with curcumin. Afterwards, alternating layers of casein and folic acid are applied. The relatively simple procedure resulted in a formulation with better cellular uptake and enhanced cytotoxic and apoptic potential.

### 4.4. Nanocarrier-Loaded Gels

**Figure 4:**
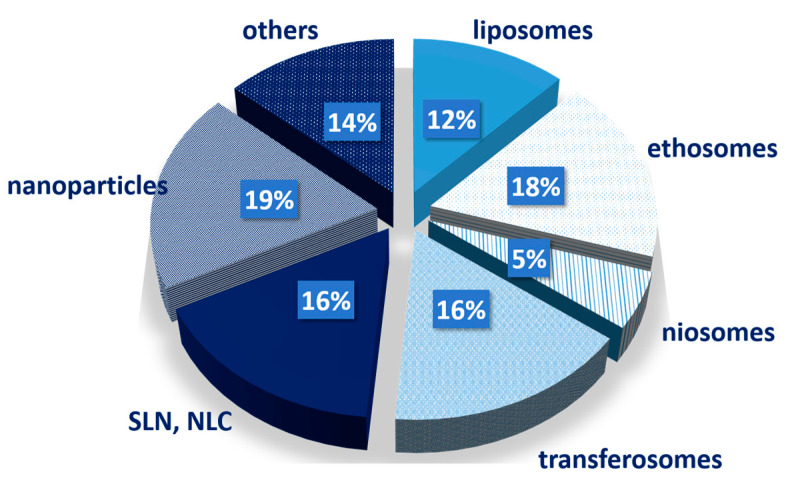
Nanocarrier-loaded gels: distribution of nanocomposites (SLN—solid lipid nanoparticles; NLC—nanostructured lipid carrier).

This type of drug delivery system simultaneously combines two technological approaches, namely, gel formulations and nanotechnology. Gel supports the nanocarriers and guarantees their application and sustained release. The nanocarriers can further provide modification of drug release due to their specific properties and characteristics. Thus, the combinatorial approach can improve the therapeutic efficacy of various drugs and routes of administration [129]. In the present review, the focus is on the topical delivery of chemotherapeutics. According to the systematic search, it was evident that for the treatment of skin cancer different nanocarriers can be loaded onto physical hydrogels. The predominant types of nanocarriers are lipid-based, as can be seen from Figure 4. This is expected due to the characteristic structure of the stratum corneum and the other skin layers.

The mechanical properties of the gel can be significantly influenced upon addition of nanoparticles, and depend on the additive concentration, modulus, and extent of additive–gel matrix interactions [130]. Therefore, in the following section the different types of nanocomposites and their effect on the formed gels are discussed.

#### 4.4.1. Nanovesicles

**Figure 5:**
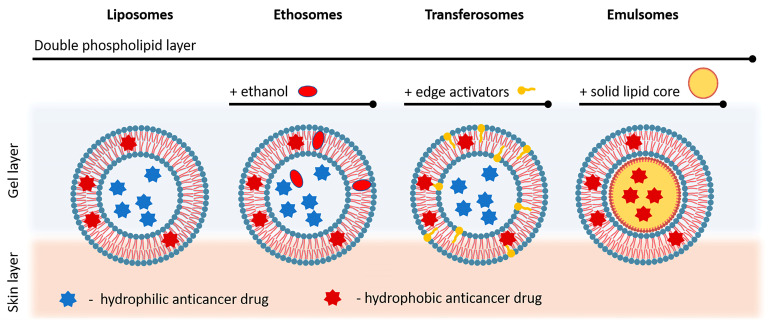
Schematic representation of nanovesicles incorporated within topical gel.

Liposomes are intensively studied nanosystems, often developed for use in anti-tumor therapy. They consist of concentric two-layer vesicles in which the hydrophilic core is surrounded by a dual phospholipid layer [131]. The drugs can be included in liposomes according to their relations, with hydrophilic drugs incorporated inside and hydrophobic in the phospholipid layer (Figure 5). Two-layer membranes can consist of natural or synthetic amphiphilic lipids and phospholipids. Liposomes can be formed with different diameters, from about 20 nm to several micrometers. The small single-layer vesicles are about 20 to 100 nm in size. They are relatively easy to prepare, with a unique size compared to other types of vesicles, and are found to be widely used. Compared to the standard formulation, some types of liposomes show a higher maximum penetration depth. The scientific literature describes various approaches in which liposomes intended for skin anti-tumor therapy are incorporated in gels that are used as suitable carriers for dermal administration.

Elastic liposomes were used by Hussain et al. as a 5-fluorouracil-delivery system in a gel based on Carbopol 980. The incorporation of liposomes in the gel resulted in a significant (double) increase in the concentrations of the drug (300 relatives to 150 micrograms) compared to the free (not gel-incorporated) liposomes. In addition, reduced side effects were observed [132]. The result clearly shows that incorporating the nanoparticles in an appropriate base such as a gel could improve the liposome nanoparticles’ advantages.

Another use related to the incorporation of liposomes in the anti-tumor therapy gel is the inclusion of different types of photosensitizers for photodynamic therapy of different types of skin cancer. Nekvasil et al. presented a preclinical study of gels containing liposomes with hydrophobic photosensitizers (meso-tetrakisporphylporphyrin or hydroxy-aluminum phthalocyanine) for photodynamic therapy in various types of skin cancers [133]. It can be concluded that the liposomal gel formulations of the photosensitizers used could be an optimum dermal preparation enabling a wide range of topical applications for numerous cancer indications, especially for nonmelanoma skin cancer. In another study, temoporfin (potent second-generation synthetic photosensitizer) is included in hydrophilic gels for photodynamic therapy with a different concentration of carbomer [134]. The resulting gels show very good stability in the studies conducted for 6 months.

Similar systems were also proposed for combination therapy, including anti-tumor agents curcumin and STAT3 Si-RNA in liposomes included in 0.4% agarose gel [20]. Studies have shown that the topical ionophoretic administration of the developed gel shows a tumor suppression comparable to the invasively applied liposomes. In addition to curcumin, in another study neringenin (an anti-tumor agent with a pronounced antioxidant effect) was included in deformable liposomes dispersed in hydrophilic gels based on hydroxyethyl cellulose (HEC) and hydroxypropyl methylcellulose (HPMC) [135]. The results show that the incorporation of liposomes in the gel leads to a further delay in the release of the drug, with a difference in release in gels based on HEC and HPMC.

Along with the inclusion of liposomes in conventional topical gels, Famta et al. included niclosamide in liposomes and incorporated it based on the Pluronic^®^ F127 and Pluronic^®^ F68 (3:1 Ratio) heat-sensitive gels characterized by the sol-to-gel transition temperature of 33 °C [136]. Their research shows that the developed compositions significantly increase the cytotoxicity of niclosamide to melanoma cells, with their subsequent inclusion in thermogel providing the ability to control release kinetics.

Other vesicular nanocarriers are transferosomes. Transferosomes are similar to liposomes in their morphology, but according to their functionality, they can deform enough to go through pores much smaller than their own size. This is due to the great flexibility of the vesicle membrane, which causes the vesicles to be highly deformable [137]. Therefore, typical transferosomes are characterized by a more elastic membrane than conventional liposomes. Another specific difference between transferosomes and liposomes is the great hydrophilies of the former, which allow the transferosome membrane to swell more than the conventional lipid vesicle bilayer [138]. Because of these advantages, transferosomes are one of the most commonly studied representatives of nanosystems used for dermal administration, with scientific data gathered regarding their inclusion in gels. One specific representative of the group is protransferosome, which is a liquid crystalline pro-ultra-flexible vesicle with a lipid structure and is in situ convertible to an ultra-flexible transferosome by absorbing water from the skin [139].

Gupta and Trivedi developed a Carbopol^®^ 940 gel, including protransferosomes containing cisplatin [140]. The authors compared the kinetics of the release and skin permeation of the prepared gel, as well as the free protransferosomes. The results show that including the vesicles in the gel improves the ability to control release and improve skin permeation. The Carbopol-based gel was used as a vehicle for transferosomes loaded with 5-FU [141], paclitaxel [142], methotrexate [143], tofacitinib [144], tamoxifen citrate [145], and others. An interesting study was presented by Shamim et al., which includes carvedilol in transferosomes for topical administration to prevent UV-induced skin cancer [146]. Using the system proposed, unwanted systemic cardiovascular effects are prevented while maintaining the efficiency of carvedilol.

Biologically active substances of natural origin are of great interest in many areas of modern pharmaceutical practice. For this reason, many scientific teams have explored the possibilities of incorporating non-synthetic substances into dermal anti-cancer systems. In the group of natural substances can be classified apigenin, included by Jangdey et al. in transferosomes [147], as well as Green Tea catechins [148], subsequently dispersed in carbomer gel. Both gel systems showed good potential for use in skin cancer. Furthermore, as has been often studied in recent years, curcumin has been used as a biologically active component in a transferosomal system included in a Poloxamer 407 gel aimed at improved skin penetration and dermal localization [149].

Another example of nanosystems often included in dermal gels for the therapy of various types of skin cancer is ethosomes (Figure 5). Ethosomes are lipid vesicular carriers formed with ethanol in relatively high concentrations, phospholipids, and water. The high ethanol concentration, which significantly distinguishes the ethosomes from other vesicles, improves skin penetration [150]. Ethosomes pass the stratum corneum to a significantly greater extent than classic liposomes [151]. The effects of combining phospholipids and a high ethanol concentration lead to greater distribution and penetration through the skin lipid pots. They are designed to improve the penetration of drugs through the skin by fluidizing the lipids into the stratum corneum.

Gels with incorporated ethosomes were prepared for dermal drug delivery of 5-FU [152], curcumin [13], vismodegib [153], brucine [15], sonidegib [154], itraconazole [155], etc. Puri et al. offer the use of ethosomes for improved dermal administration of 5-FU, thus overcoming the limiting factors of the drug, such as limited skin permeation, retention at the target site, and possible skin irritation [152]. Ethosomal systems are subsequently included in Carbopol^®^ 934P-based gel and have been tested in terms of skin permeation and deposition, showing significantly increased skin permeation (5.9 to 9.4 times) and anti-tumor potential compared to cream (commercial product), in addition to reduced skin irritations. The possibility of incorporating ethosomes loaded with vismodegib into the carbomer gel has also been studied, providing data on increased anti-tumor activity, compared to oral drug forms of the same active substance [10,153]. Similar results (threefold increased bioavailability and significantly increased anti-tumor activity) have been observed by other authors regarding the drug sonidegib, included in the ethosomes system in the gel based on carbomer [154]. The same polymer has also been used in the inclusion of fisetin-containing binary ethosomes, with the results being positive in terms of achieving better release control and improved skin penetration compared to a conventional gel containing the same drug [156].

Although most ethosomes tests have been conducted in carbomer-based gels, a study by Abdellateif et al. displays better controlled drug release from a hydroxypropyl methylcellulose (HPMC) 2.5% *w*/*w* gel system, containing ethosome-loaded celecoxib, compared to the same ethosome dispersion [157]. In the same study, the data were compared to the carbomer-based gel, taking into account different release kinetics depending on the polymer used.

Another type of nanovesicle is the emulsomes (Figure 5). These were used by Sahu et al. as methotrexate carriers and were further incorporated in a Carbopol^®^ 934 gel for the management of skin cancer through topical delivery. The gel shows a delayed release of the drug for a period of 12 h, as well as a good profile of stability, examined during 45 days period of testing [158]. In addition, a different type of hydrogel containing vesicular structures—glycerosomes loaded with plumbagin—showed increased anti-tumor activity and improved skin penetration [159].

#### 4.4.2. Lipid Nanoparticles

In recent years, lipid nanoparticles have been of great interest in the field of pharmaceutical technology. They are colloidal carriers, characterized by a solid lipid core consisting of a mixture of hard and liquid lipids and having particle dimensions in the nanometer range. They consist of a lipid matrix with a special nanostructure. These nanostructures improve the drug loading and firmly retain the drug during storage. Nanostructured lipid carriers weaken the barrier function of the stratum corneum and improve the penetration of drugs through the skin. There are data on topical gels containing lipid nanoparticles for the skin drug delivery of silymarin [160], topotecan [161], and others. The prepared lipid particle-containing gels are characterized by improved anti-tumor activity after dermal administration.

EL-Sheridi et al. used the antifungal agent with good anticancer activity, itraconazole (ITC), unmodified or modified with the amphiphile miltefosine or the lipopeptide biosurfactant surfactin lipid systems for low-risk cancers of the skin [162]. The prepared lipid nanosystems were subsequently included in the gel, comparing the tumor-inhibitory capacity of a conventional gel with itraconazole and the gel-containing nanoparticles, with better results achieved with the gel containing lipid nanoparticles.

Khallaf et al. showed that 5-FU-loaded SLNs (mean size of 137 ± 5.5 nm and zeta potential of −19.70 ± 0.40 mV) based on lecithin and poloxamer 188 could deliver the drug topically and treat tumors when loaded on a negatively charged physical hydrogel vehicle (CMC). The results show that a repulsion of charged particles and the gel charges may be the reason for higher dermal permeability [163].

Some authors expanded the studies of gels loaded with lipid nanoparticles, and offered dual load systems, aiming to synergize the effect of the biologically active substances involved. As an example, there is the simultaneous inclusion of cannabidiol and 5- fluorouracil in lipid nanoparticles, followed by incorporation in a gel based on Carbopol^®^ 934 [164]. The dermal administration system shows a good toxicological profile and improved penetration through the skin barrier compared to conventional drug delivery systems. Other examples of similar systems are lipid-based gel for the co-delivery of quercetin and resveratrol [165], and 5-FU and resveratrol [166].

#### 4.4.3. Inorganic Nanoparticles

**Figure 6:**
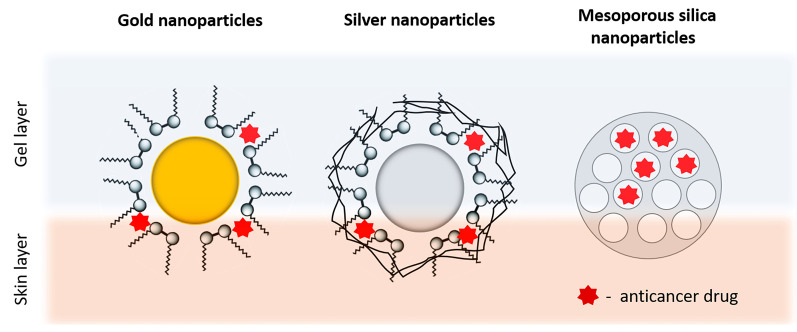
Schematic representation of inorganic nanoparticles incorporated within topical gel.

Inorganic nanoparticles are presented by drug delivery systems based on different inorganic materials such as metals, silica, and carbon materials (Figure 6). Metal nanoparticles, most often silver (AG) and gold (AU), are examined as potential transdermal carriers due to their easy preparation, ability to modify their surface, and adjustable size. It is assumed that the transdermal transport of gold nanoparticles is related to their ability to interact with skin lipids by changing the stratum corneum by inducing transitional and reversible openings. One of the fastest developing areas in the field of inorganic nanoparticles is the development of mesoporous silicate nanoparticles. The key properties of this versatile material are due to the arranged structure of the pores (sizes from 2 to 50 nm) and surface chemistry, which allows insertion in a controlled manner of new functional groups [167]. The interest in these nanoparticles is explained by their high specific surface area (>1000 m^2^/g) and large volume (~1.0–2.0 cm^3^/g), as well as the high capacity of sorption of drug molecules, the relatively simple, cheap, environmental, and controllable procedure of synthesis, their biocompatibility, and their lack of toxicity. Mesoporous silica particles have been shown to increase the aqueous solubility of poorly water soluble drugs, which significantly improves their transdermal administration [168].

Safwat et al. used gold nanoparticles covered with CTAB (cetyltrimethyl ammonium bromide) as a possible carrier for 5-FU. The prepared nanocarriers with a mean size of 16.02 ± 0.22 nm and positive zeta potential (+47.81 ± 0.43 mV) were additionally dispersed in the Pluronic^®^ F127 gel for topical application on tumor xenografs [6]. The gel thus obtained showed twice as high permeability through mice skin compared with conventional 5-FU gel. Capanema et al. synthesized hydrogel for doxorubicin delivery based on embedded silver nanoparticles within cross-carboxymethylcellulose [113]. Interestingly, in this case, a green process involving the in situ reduction of Ag+ by the polymer, followed by the electrostatic conjugation with DOX, was used to form colloidal nanocomplexes. The nanostructures obtained were further chemically cross-linked using citric acid under mild conditions. The predecessor of mesoporous silica—inorganic zeolites—have been used as a skin delivery system for carmustine after their inclusion in the HPC gel [169].

#### 4.4.4. Other Nanocarriers

Nasr et al. studied gel formulation based on 2% Carbopol^®^ 934, including ethosomes and lipid-coated chitosan nanocarriers loaded with ferrous chlorophyllin, designed for photodynamic therapy for squamous carcinoma [170]. Sun et al. reported a better effect of curcumin-loaded PLGA included in the gel compared to free-drug-loaded hydrogel in a mouse model [103]. The carboxymethylcellulose polymer nanoparticles included the anticancer drug doxorubicin. Specifically in this case, the polymer available in the synthesized nanocomposites was further chemically modified to obtain a hydrogel showing good effectiveness against melanoma cancer cells with reduced toxicity to healthy cells [114].

A dual mucoadhesive topical gel containing gellan gum nanoparticles with cisplatin and liposomes with paclitaxel was prepared by Bhardwaj et al. [171]. Interestingly, the gel was prepared after dispersing the pre-prepared nanoparticles in the high acyl gellan solution.

Chitosan hydrogel was used in the system consisting of the immune adjuvant imiquimod in the form of polydopamine-coated nanocrystals in order to achieve an immunomodulation effect as well as immunogenic cell death induction [172]. In addition, using the prepared system resulted in inhibited melanoma growth and metastatic processes.

A new drug delivery system for the local treatment of dermal cancer, based on highly elastic and deformable nanostructures with a size of about 100 nm and positive zeta potential, was proposed to enhance the skin therapy with an antioxidant diindolylmethane derivative [173]. The authors hypothesized that the positively charged nanostructures could interact with the stratum corneum due to its negative charge. Based on the results, it was also suggested that incorporating the nanovesicles in a hydroxypropyl methylcellulose physical hydrogel may contribute to its increased deposition within the skin. The hydrogel probably leads to hydration and disruption of the stratum corneum, followed by an opening of channels within the lipid lamellae domain. The nanoparticles could then use the gap to penetrate into the skin.

### 4.5. Other Gels

Here, some other types of gels were identified that do not belong to the previously discussed classes, as can be seen from the examples in Table 3. The search found 10 articles that discuss nanoemulgels, bigels, emulgels, or microgels, or in which the type of gel is not clear due to the unavailability of the full texts. Microgels can be defined in a similar way as nanogels, but the difference arises from their bigger sizes, which fall into the range of 100 nm to 1 µm [70]. Even though clear definitions based on the particle size exist for the macrogels, microgels, and nanogels [70], it can be seen that different researchers apply the terms interchangeably to some extent (Table 2 and Table 3), and these size ranges are only approximate.

One example is the microgel prepared by Puga et al. based on chitosan and cross-linked with glutaraldehyde with particles in the size range 200–600 µm [174]. This proposed formulation is suggested for oral or dermal delivery of the chemotherapeutic agent 5-fluorouracil. They showed pH-dependent release, with delayed release of an acidic medium with a limited burst effect due to the pectin coating. This study showed promising results as a topical treatment of malignant melanoma.

Bigels are intimate hydrogel/oleogel colloidal mixtures in which each of the corresponding liquid phases is independently stabilized by means of a suitable gelling agent. One significant advantage of these gels is the decreased expenditure required for preparation in comparison to the formulation of gels containing liposomes or other nanoparticles and nanovesicles [70]. In this review, one article was identified dealing with the preparation of a bigel for topical delivery of imiquimod [175]. This study investigated the possibility of dissolving the chemotherapeutic drug in fish oil, gelling it with beeswax and mixing it with previously prepared physical carbomer hydrogel. The results showed that the skin permeation was superior in a simple oleogel, and in the bigels, it depended on the ratio between the two corresponding gels. It was found that the fatty acids in the fish oil modify the stratum corneum permeability for the lipophilic API. Once again, it was confirmed that the drug’s solubility and the stratum corneum’s barrier function are of utmost importance for the depth of drug delivery. At the same time, the rheological and mechanical characteristics of the bigels are better than those of an oleogel. It can be said that a compromise needs to be met between the ideal penetration and optimal application formulation.

As shown by different researchers, incorporating lipophilic APIs in various lipids can improve their solubility and permeability [17,18,176]. Nagaraja et al. developed an emulgel for the delivery of chrysin, a phytochemical with broad therapeutic activity but with limited solubility and bioavailability [17]. Emulgels are emulsion-based gels and can be of the type o/w or w/o emulsion that is further gelled by a suitable gelling agent [70]. Their properties provide them with significant advantages for the dermal application of drugs. Some of the most important are the thixotropic behavior, spreadability, lack of greasiness, emollient action, and longer shelf life. The approach for improving drug delivery to cancerous skin proposed by Nagaraja et al. [17] proved to be versatile for hydrophobic constituents. It leads to a decrease in overall dose and thus can guarantee lower systemic penetration and limit the associated side effects.

## 5. Methods for Characterization of Gels

Gels, as presented above, can be synthesized from various materials—organic, inorganic, and hybrids. Moreover, they can vary from semi-fluid and semisolid to solid-like substances such as hydrogels or xerogels, which form a powder after drying. Therefore, various techniques are used to characterize gels’ chemical and physical structure, morphology, and overall properties, as well as rheology. A summary of the characterization techniques is presented in Table 4, and these are briefly discussed in the following subsections.

**Table 4:** Summary of methods for gel characterization and their application.

Type of Characterization	Method	Application
Physico-chemical characterization	X-ray	The method is used for determination of the crystalline structure of polymers including crystal size, crystalline phases and their amount, crystallinity level and texture [177,178,179].
	pH	Gel sample is diluted in purified water to obtain a concentration of 10% w/v. A potentiometric measurement of the pH could be used to elucidate skin tolerability and possible stability issues [106].
	UV-vis spectroscopy	It can be a useful tool for studying functional groups present in chemical gels, or molecular arrangement, such as π stacking, between aromatic rings in physical gels [179,180,181]. It can be also applied for the determination of lower critical solution temperature (LCST) related to the sol–gel transition properties [182]
	Infrared (IR) spectroscopy	IR spectroscopy is one of the most used confirmation methods to prove the structure of newly synthesized or already known polymers as a basis for gel preparation [13,17,18]. New functional groups or their absence are accounted for based on the bond energies that can be determined by the method [180]
	Nuclear magnetic resonance (NMR)	The specific transitions detected in the peaks can imply what kind and how many atoms are there in the structure. The chemical shifts are dependent on temperature and concentration, and thus, the NMR technique can be applied in gel characterization. Molecules come closer together in more concentrated solutions and this fact can be used to investigate the sol–gel transition in gel formulations [183]
Thermal characterization	Thermogravimetric analysis (TGA)	In case of gels this method presents information regarding the thermal stability and the phase transitions of the gels [184,185].
	Differential scanning calorimetry (DSC)	It can be used to determine compatibility between various polymer blends, interaction of the gelling agent with the API, thermal stability, etc. [99]. It can be also applied to determine the interaction of the skin with the formulation as the thermogram of untreated skin shows one endothermic peak at 78 °C due to melting of stratum corneum lipid. If interaction is observed there would be a change in this peak [185].
Morphological characterization	Scanning electron microscopy (SEM)	Dried gel samples can be observed for their surface morphology at significant magnifications (300,000×). In addition, the high resolution allows detection of nanostructures with the gel base [186]. The data about surface roughness can be related to cell adherence and sustained drug release [186]. A confirmation of size could also be derived from this method [126]
	Transmission electron microscopy (TEM)	The technique could be used in the chemical hydrogels and nanogels characterization, providing a very thin layer can be produced; otherwise, they are not visible on TEM [187].
	Atomic force microscopy (AFM)	Direct observation of gel surface in water is possible providing specific conditions of the measurement are selected [188]. Usually the method is performed in non-contact mode for the evaluation of surface roughness. Furthermore, using force–distance curves, the elastic modulus of the sample can be determined. Adhesion to substrates (such as cells) can be another parameter determined by the method [189].
Mechanical characterization	Viscosimetry	Gel formation can be measured by monitoring their elastic (G′) and viscous (G″) moduli. When the value of G′ exceeds the one of G″, a gel is formed [190]. Furthermore, the gel kinetics can be observed [22].
	Texture analysis	The method allows evaluation of various parameters such as cohesiveness, adhesiveness, hardness, and extrudability [106,175]. They determine the ability to spread on the skin, the bioadhesion, and the ability to be evacuated from the packaging [106].
	Spreadability	It is evaluated by the parallel plate method. A definite amount of the gel is placed on a glass plate. Another plate with known weight is positioned on top. After predetermined intervals additional weights are applied. The radius of spreading is measured and further used to calculate the spreadability factor [106,191,192].
Performance characterization	Swelling	Test for the ability of chemical hydrogels and nanogels [14] to imbibe water upon immersion in different liquid media (deionized water, phosphate buffer with various pH). The increase in weight of the gel sample over time is a measurement of its swelling ability. The results are usually directly related to the release of the loaded API [14,126] and the mechanical stability of the gel [114,193].
	Occlusion in vitro	A beaker is filled with water, covered by cellulose acetate filter, and sealed with Teflon tape. A predetermined amount of the gel formulation is evenly placed on top. These samples are kept in skin-mimicking conditions (temperature 32 °C) and constant humidity for 48 h. As a reference, a sample covered with filter paper only is used. The occlusion factor is calculated based on the change in weight of the samples [83,194,195,196].
	Occlusion in vivo	The test is performed on healthy volunteers with an established protocol of application. At the beginning and after one week of application the skin hydration is determined based on capacity measurements with specific probes of Soft Plus apparatus (Callegari Srl, Parma, Italy) [196,197]
	In vitro release	This is an acellular assay in phosphate buffer with different pH [114] through a dialysis membrane [157] or with a membrane attached to a Franz-diffusion cell [137,143] which measures the amount of drug released over time.
	Permeation studies	Ex vivo hairless animal skin is used together with a Franz-diffusion cell to measure the amount of drug that passes through the membrane [38,123,124]. Artificial membranes can be applied such as Strat-M^®^ membrane or keratinocytes culture (EpiDerm^TM^) in a Franz-diffusion set-up [80]. They are standard and thus provide better reproducibility of the results [198,199].
	Skin deposition studies	Confocal laser scanning microscopy (CLSM) is used in combination with permeation studies in order to evaluate the depth of penetration, cell internalization, and formulation factors affecting them. Usually, rhodamine B or other fluorescent dye is loaded in the tested sample. A substrate is treated with the gel formulation and optically scanned with fluorescent microscope. The substrate could present in vitro grown cells [156], excised skin sample used in a Franz-diffusion set-up [165,200], or in vivo observed skin [201] Tape stripping technique can non-invasively measure the amount of drug penetrated in the stratum corneum [201,202,203].

### 5.1. Physico-Chemical and Thermal Characterization Methods

The complex nature of the topical gels is reflected in a significant number of parameters that can be evaluated. The most important ones based on their frequency of utilization and informative value for dermal gels are summarized in Table 4. It can be seen that there are various aspects regarding the chemical, physical, morphological, and performance properties of the topical formulations. Some of the methods are well-established techniques (X-ray diffraction, UV-vis and IR spectroscopy, NMR, pH measurement, TGA, DSC, etc.) applied in the characterization of diverse materials and nanoformulations including gels. There are current reviews that elucidate in more details the principles and application of those methods [204,205]. As the focus of the current review is on topical administration of the gel-based formulations, more details are discussed about the mechanical and performance characterization techniques that are directly related to their application.

### 5.2. Rheology Studies

Gels possess specific properties from a physical point of view as they are neither solid nor liquid. Their behavior is complex; therefore, the deformation and flow pattern are studied by rheology. Viscosity can be affected by various factors including polymer concentration, pH and ionic strength of the medium, temperature, and particle size of the polymer [22]. Its evaluation is of paramount significance as it provides information regarding the ability of the gel formulation to spread on the skin. The drug release is also affected by the rheological behavior of the gel [141,175,176]. Most of the performed studies indicate that gels are non-Newtonian systems and possess pseudoplastic rheology [100,111,191]. This indicates that the viscosity decreases in high shear, thus facilitating the gel application. Typical yield stress for semisolid formulations is in the range 20–50 (80) Pa and varies according to the method used for its determination [153]. The incorporation of cyclodextrin complexes within the HPMC gel matrix could lead to a decrease in the apparent viscosity, possibly due to hydrophobic interactions between the cyclodextrins and the polymer chains [97]. The composition and type of liposomes also affect, in a different manner, the rheology behavior of a carbomer gel, and can either increase or decrease its yield stress, apparent viscosity, and elasticity [206]. In the case of ethosome-loaded carbomer gels, a slight decrease in viscosity is observed, most likely due to the presence of ethanol in the nanovesicle structure [153,154,207]. On the contrary, the loading of a hydrogel with NLC leads to an increase in the viscosity in comparison to a corresponding gel with no nanostructures embedded within it [44]. Therefore, the incorporation of nanoparticles may affect the rheological behavior of the gel, and different release and skin retention can be observed. The effect of the nanocomposites in gels is not always studied in the articles identified in the current review. It would be beneficial to compare the conventional gel and the nanocarrier-enriched gel in terms of viscosity, as this is directly related to the applicability of the formulation and the drug release.

### 5.3. Morphology Charcaterization

**Figure 7:**
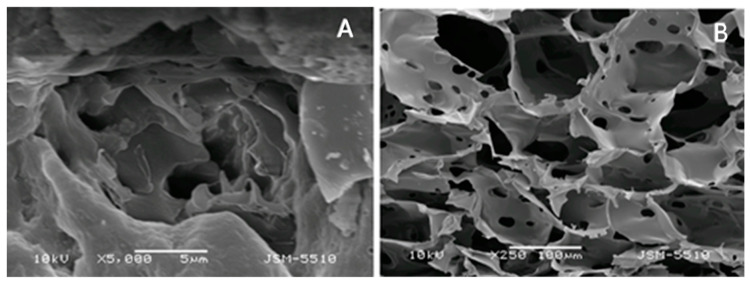
SEM images showing the application of the technique for the evaluation of hydrogel morphology: (**A**) polymer powder; (**B**) lyophilized gel sample at ×250 magnification.

Gels’ morphology can be studied by transmission electron microscopy (TEM) and scanning electron microscopy (SEM). They can be used to study amorphous, crystalline, organic, and inorganic specimens. Examples of the observed differences in morphology of a chemical hydrogel can be seen in the images taken with SEM and presented in Figure 7. The images were obtained in another study of ours and have not been published elsewhere. The polymer’s morphology itself (Figure 7A) differs from the appearance of the polymer in gelled form. Figure 7B shows the lyophilized hydrogel and there is the clear presence of large interconnected pores 

Das et al. [208] obtained cryo-SEM micrographs of three samples of successfully grafted hyperbranched poly(acrylic acid) chains from the surface of guar gum. They found deformations in their structures in the swollen conditions compared with the dried samples’ SEM images. The authors expect the pores in these samples to enlarge upon swelling in water. It is a common phenomenon that, whenever a hydrogel swells in an aqueous medium, the polymer deforms, i.e., the pores in the polymer become bigger and distorted. Typically, in a cryo-SEM image that captures the cross-section of a frozen swollen polymer, deformation of the pores is depicted.

### 5.4. Performance Characterization

The performance characterization is related to the properties of the formulations directly affecting their ease of administration, residence time on site of application, release of the active constituents, and permeation through the skin and reaching the site of action. These settings are discussed in the following subsections.

#### 5.4.1. Occlusion

The occlusion properties of any semisolid formulation are of compelling significance for the normal transepidermal water loss of human skin. It is directly related to the skin hydration and especially that of the stratum corneum. Consequently, the occlusion effect can promote the percutaneous absorption [83,194,195,196,209]. At the same time, occlusion may produce unwanted effects. In the case of transferosome-loaded dermal gels, it was established that the transepidermal osmotic gradient is crucial as a driving force for the elastic transport into the skin [210]. Some adverse effects may also be attributed to significant occlusion [211]. The occlusion factor is strongly dependent on the crystallinity in the case of solid lipid nanoparticles or nanostructured lipid carriers [212]. The data suggest that the higher the crystallinity level, the higher the occlusion factor.

#### 5.4.2. In Vitro Release

The in vitro release can be an acellular technique, i.e., a dissolution test study with proper modifications such as applying membranes between the donor and receiver compartment. It is applied for a preliminary evaluation of the properties of the formulation and the factors affecting them. The conditions of the experiment should mimic the physiological ones of the administration route. This means that a temperature of 32 °C and pH of the medium between 5.0–6.0 would ideally be used to reflect the healthy skin conditions [213]. Nevertheless, the results of the current review show that various parameters of the release set-up are applied, i.e., varying temperatures: 25° [137], 32° [4], and 37 °C [4,143,144,157] and varying pH values: 5.5 [4], 6.8 [137,157], and 7.4 [4,143,144]. In addition, the medium can contain surfactants such as Tween 80 [137] or sodium lauryl sulfate [157] to maintain sink conditions. These variations in performing the tests yield different results which are hard to compare in terms of efficiency of the proposed formulations.

#### 5.4.3. Drug Penetration

Evaluation of the drug level within the skin (penetration evaluation) plays a key role in the formulation of transdermal and topical drug dosage forms. Depending on the conditions, drug permeability study can be performed in vivo, ex vivo, and in vitro.

##### In Vivo Methods

The in vivo methods can provide the most accurate information regarding the drug penetration. Nevertheless, those methods are characterized by complexity and variability, and require living species. The “gold standard” in determining drug penetration through the skin is by application of the tested dosage form to a particular subject and evaluating drug levels on the target skin tissue [202]. Unfortunately, clinical trials are too expensive and can lead to harmful outcomes. The in vivo methods can be further classified as invasive and non-invasive, as the latter are preferable [214].

##### Pharmacokinetic Studies

Pharmacokinetic studies are of high importance for the evaluation of transdermal delivery since they can fully resemble the processes in the human organism. In general, the pharmacokinetic evaluations use the drug’s plasma concentrations and the parameters of interest such as the area under the curve (AUC) and C_max_. Unfortunately, detection of these parameters after topical and transdermal delivery is not always possible due to the very low concentrations achieved [202].

##### Skin Biopsy and Suction Blister Methods

Skin biopsy and suction blister are invasive methods for evaluation of drug penetration [202]. Even though they give precise information of drug deposition in skin, due to the tissue damages they are not a preferable approach for a regular evaluation of skin penetration [214].

##### Microdialysis

The microdialysis technique is a less invasive (semi-invasive) in vivo technique that measures the drug concentration directly in the target tissue, giving full information about the drug penetration through the skin [215]. This method uses a semi-permeable tube (catheter), representing a dialysis membrane, inserted underneath the skin [216]. After application of a dosage form on the skin, the API passes through its layers, reaches the catheter and accumulates in the perfusate. The samples are taken at different time intervals and the amount of drug is measured by the usual analytical methods [214].

##### Tape Stripping

**Figure 8:**
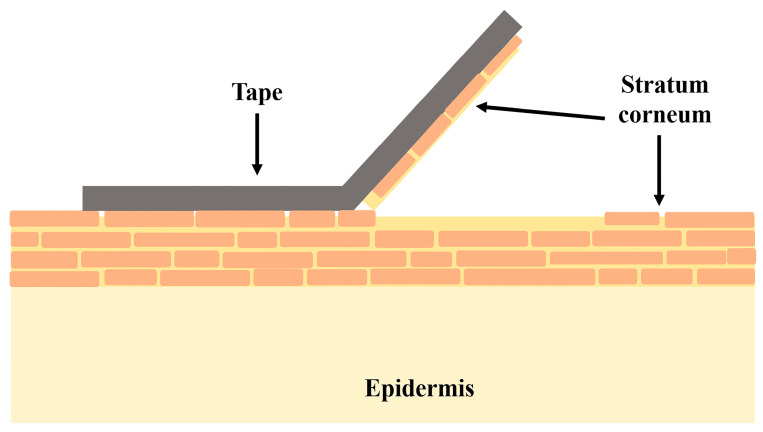
Schematic representation of tape stripping technique.

Tape stripping is an inexpensive, efficient, quick, and minimally invasive technique used to determine the skin penetration and bioavailability of topically applied drugs whose target is viable skin tissue [201,202,203]. This procedure can be performed in vivo and ex vivo using skin model membranes instead of human volunteers and animals. The method includes repeated application and subsequent removal of an adhesive film on the skin surface after administration of the tested topical dosage form [215] (Figure 8). Upon removal, cells from the stratum corneum are also removed. The sample contains corneocytes and a certain quantity of drug molecules, which can be extracted by selective solvents and determined by the classical analytical method [203]. In order to achieve representative results, all remnants of the applied formulation should be removed. Therefore, the first piece of tape is discarded [215,216]. Moreover, the tests should be performed under the same conditions, regarding skin area and velocity of tape removal [214].

##### Confocal Laser Scanning Microscopy (CLSM)

Unlike the previously mentioned techniques, confocal laser scanning microscopy (CLSM) is a non-invasive method that uses fluorescence phenomenon in order to examine the drug distribution throughout the skin [215]. Usually, rhodamine B or other fluorescent dye is loaded in the tested sample. A substrate is treated with the gel formulation and optically scanned with a fluorescent microscope. Therefore, CLSM has potential in analyzing the localization of different nano- and micro-sized structures in the skin delivery. However, the main disadvantage of this technology is the limited number of fluorescence markers [214].

##### In Vitro Methods

Even though in vitro assay cannot completely resemble the complexity of the human skin structure, it is an essential tool for assessing drug release behavior as well as measuring in vivo skin absorption [215].

**Figure 9:**
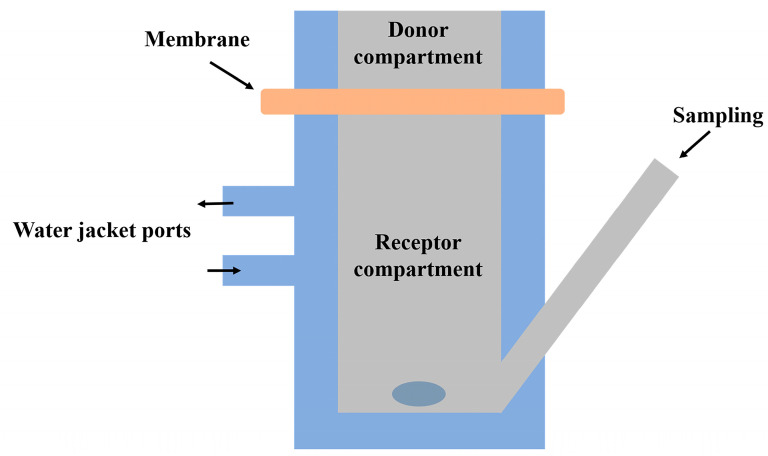
Schematic representation of Franz-diffusion cell.

The Franz-diffusion cell is well-established experimental set-up with a structure designed to mimic skin conditions [217]. This tool consists of a cell that holds a chamber for drug application (donor compartment), a membrane within which the drug can diffuse, and an acceptor media chamber (receptor compartment) from which samples can be collected [201] (Figure 9). Measurement of the amount of drug into the receptor compartment by a suitable analytical method is used to evaluate the drug diffusion in the skin over time [214].

Different techniques evaluating the API permeation usually estimate the drug flux, which is expressed as the amount released through a definite surface area over time (mg/cm^2^/h). Based on the flux value, the permeability coefficient can also be determined, taking into account the drug concentration in the donor compartment [143]. These set-ups utilize Franz-diffusion cells with a membrane separating the donor and acceptor compartments. The membrane can be either an excised hairless animal skin (such as goat [143], rat [12,218], or pig [38,84,97,219]) or an artificial skin (StratM^®^, EpiDerm^TM^). The choice of the media for the receptor compartment depends on the solubility of the API, but aqueous buffers with physiological pH values are preferred. To increase the API solubility and achieve sink conditions, surfactants (such as Tween) and co-solvents (such as ethanol) are also allowed to be added. The factor that has the strongest influence on accurate and representative results is the choice of an appropriate membrane [201]. Even though human skin is the most relevant model for the evaluation of in vitro skin permeation studies, its use is usually replaced by animal models such as pigs, rats, mice, and snakes [220]. Among these, porcine ear skin is thought to most resemble normal human skin, due to its thickness, follicular structure, vascularity, and lipid content [38,84,97,219]. Unfortunately, due to ethical considerations, today there are many regulatory restrictions on the use of animals [216,221]. For these reasons and taking into account the advances in tissue engineering, the development of artificial in vitro human skin models that resemble both healthy and diseased skin have been explored widely [216,220]. The artificial skin membranes can be made of simple homogeneous polymer materials, such as poly(dimethoxysilane), silicone membranes, or even lipid-based parallel artificial membrane-permeability assay (PAMPA) or phospholipid vesicle-based permeation-assay membranes [216]. Their main advantage is their easy reproducibility, which allows studying the basic mechanisms controlling skin permeation [221]. Moreover, they can be modified according to the acquired specific disease characteristics. For example, skin cancer models were constructed by incorporating various tumor entities within the three-dimensional (3-D) matrix, including cultured melanoma cells, melanoma tumor spheroids, and various cutaneous squamous cell carcinoma cell lines [220].

The controversy surrounding all characterization methods and the information they provide once again prove the complexity of the dermal route of administration. The skin is an easily accessible and non-invasive application site. However, the skin’s properties, together with the effects of the formulations on the skin, explain the intensive scientific research in the area and the continuously growing number of research articles.

## 6. Conclusions and Future Aspects

The current review evaluated the achievements and hurdles in the development of suitable topical formulations for the delivery of chemotherapeutic substances of various properties in the treatment of skin cancer. The review showed the complexity of the matter. Knowledge from a large number of scientific areas is needed in order to be able to prepare and characterize an efficient drug delivery system. It is evident that a large amount of scientific effort has been put into improving the penetration, stability, and efficacy of the APIs. Various approaches have been implemented, such as the addition of penetration enhancers, preparation of different types of gel carriers, loading with nanocomposites, and other strategies. However, the results from the current review showed that there is a wide variety of terms used to classify these complex systems. The lack of clear, uniform, and accepted terminology makes investigating and understanding the mechanisms behind some of the findings difficult. Therefore, in our opinion, the general classification of gels used in this article could be convenient for future studies and distinguishing between different formulations. It can also be noted that a significant number of characterization tests exist for the explanation of the gels’ various properties. There are a large number of variations in the penetration tests, and the results from in vivo, in vitro, and ex vivo studies vary. This is most likely due to the skin’s complex structure and physiological properties. Therefore, the mechanism by which these processes occur requires further clarification. Nevertheless, it is possible to deliver both hydrophilic and lipophilic drugs by combining the nanotechnological formulations together with the gel supporting system. The latter can provide an easy, acceptable, and versatile carrier in which various nanoparticles and nanovesicles can be loaded.

## Figures and Tables

**Table 2:** Examples of APIs formulated in nanogels and the corresponding excipients used.

Composition	Additives	API	Particle Size [nm]	Specific Features and/or Comments	Ref.
PLGA ^1^ (0.1%), chitosan (0.4%), Poloxamer 108 and 407, PVA ^2^ (0.4%)	Eucalyptus oil coating	5-fluorouracil	190–220	-	[38]
Chitin	^-^	5-fluorouracil	125–140	-	[124,125]
Cross-linked chitosan Poloxamer 407		Bleomycin	140–170	pH sensitive	[126]
Chitosan, TPP ^3^, Poloxamer 407	Transcutol^®^ coating	Capecitabine	120–160	pH sensitive	[123]
Chitosan, TPP ^3^, Poloxamer 407	Transcutol^®^ coating	Temozolomide	170–200	pH-sensitive	[43]
Deacetylated-β-chitosan grafted with ρ-coumaric acid	^-^	*Syzygium aromaticum* essential oil	200–460	Newly extracted squid β	[127]
CMC ^4^-casein	Casein and folic acid coating	Curcumin	-	Layer by layer coating; folic acid active targeting	[14]

^1^ PLGA—Poly(lactic-co-glycolic acid); ^2^ PVA—Polivinylalcohol, ^3^ TPP—sodium tri-polyphosphate; ^4^ CMC—carboxymethyl cellulose.

**Table 3:** Examples of APIs formulated into other types of gels and the corresponding excipients used.

Type of Gel	Gelling Agent	API	Particle Size [nm]	Specific Features and/or Comments	Ref.
Microgel	Chitosan coated with pectin	5-fluorouracil	200–600 nm	pH-sensitive	[174]
Bigel	Carbopol^®^ 940 (3%) and beeswax (10%)	Imiquimod	-	Hydrogel + oleogel based on fish oil mixed at 50:50 ratio	[175]
Nanoemulgel	Poloxamer 407 (20%)	Chrysin	157 nm	Self-nanoemulsifying preconcentrate was further dispersed in gel	[17]
Nanoemulgel	Protasan™ UP G 213	Daidzein	190–210 nm	Nanoemulsion-based gel	[18]
Nanoemulgel	CMC (3.5%)	*Mentha spicata* essential oil	189–464 nm	Gelled nanoemulsion	[176]

## Data Availability

Not applicable.
